# Can Early Life-Stages of the Marine Fish *Sparus aurata* be Useful for the Evaluation of the Toxicity of Linear Alkylbenzene Sulphonates Homologues (LAS C_10_-C_14_) and Commercial LAS?

**DOI:** 10.1100/tsw.2002.222

**Published:** 2002-06-20

**Authors:** M. Hampel, I. Moreno-Garrido, J. Blasco

**Affiliations:** Instituto de Ciencias Marinas de Andalucía (CSIC), Campus Río San Pedro, 11510, Puerto Real, Cádiz, Spain

**Keywords:** linear alkylbenzene sulphonates, anionic surfactants, wastewater, early life-stages toxicity test, LC_50_, seabream, larvae, eggs

## Abstract

Most commercial household cleaning agents and personal care products contain the anionic surfactant linear alkylbenzene sulphonates (LAS) as the active compound. After their use they are discharged, theoretically after adequate wastewater treatment, into receiving waters finally reaching estuaries and coastal waters. Laboratory toxicity tests are useful tools in determining at which concentration a certain wastewater compound becomes hazardous for an existing group of organisms. Early life-stage toxicity tests include exposure during the most sensitive development period of the organism. In fish, this type of assay has shown to predict accurately maximum acceptable toxicant concentration (MATC) values (comprised in the range defined by the NOEC and LOEC) in fish early life-stage tests. For this reason, larvae of the seabream, Sparus aurata, were exposed to increasing concentrations of LAS homologues (C_10_-C_14_) and commercial LAS. Obtained LC_50_ values ranged between 0.1 and 3.0 mg l and were compared with LC_50_ values of previous hatching experiments with the same species. Larvae proved to be more sensitive to LAS exposure of individual homologues than eggs, except in the case of commercial LAS. LC_50_ values can be directly employed to determine their potential risk in a concrete environment with known pollutant concentrations. Dividing the LC_50_ value with the found homologue concentration and extrapolating with certain security factors proposed by different environmental organisms, potentially hazardous pollutant concentrations may be detected. Average estuarine or coastal LAS concentrations are generally below toxicity limits for this kind of organism, considering that the average alkyl chain length of commercial LAS is 11.6 carbon atoms.

## INTRODUCTION

Linear alkylbenzene sulphonates (LAS) are the most commonly used surfactants in industrial and household cleaning agents. In 1998, 2.8 million metric tons of LAS has been consumed worldwide after being discharged into receiving waters, and finally reaching estuaries and coastal waters[1]. After adequate wastewater treatment, 95 to 98%[2] of LAS is generally removed, whereas receiving water concentrations of LAS should be generally below chronic NOECs reported for fish, algae, and invertebrates. Nevertheless, EU wastewater treatment legislation is implemented slowly in Member States and a large number of important European cities still do not comply with the obligatory facilities. Even though a considerable number of the 542 large European cities of more than 150,000 inhabitants are equipped with adequate treatment, 37 are discharging all their wastewater into the environment without prior treatment, and 72 discharge a large proportion of their wastewater into the environment[3] with inadequate or no treatment. Biodegradation of LAS continues in receiving freshwater and marine systems resulting in further reductions of exposure concentrations[4,5]. While the average residence time of LAS in freshwater is about 3 days, in marine waters its half-life is between 6 and 9 days[6].

LAS has shown to interact with biological tissues, and concretely in fish, surfactants can produce damage in the gills, skin and pharynx[7,8]. Apart from the damage in the organs which are in direct contact with the polluted environment, these compounds can penetrate into the organism and exert adverse effect on the internal functions, such as inhibition of the activity of antioxidant enzymes such as catalase or superoxide dismutase[9]. In aquatic toxicity testing, fish eggs and larvae are attracting considerable interest, particularly because the developing fish embryo or larvae is generally considered the most sensitive stage in the life-cycle of a teleost[10,11].

In order to manage wastewater discharges into the environment, $LC_{50}$ values of the most sensitive organism forming part of a concrete environment should be known for the most important pollutants in wastewaters in order to guarantee the conservation of the environment. The most sensitive organisms are, generally, early development stages or organisms from the first step of the trophic chain such as microalgae and early development stages of crustaceans, fish, molluscs, etc. whose survival in the ecosystem is essential for the persistence of the ecosystems. In the last 25 years, toxicity tests with early life-stages of organisms have attracted more and more interest in aquatic toxicity, due the fact that, although they do not provide information about the effects produced by exposure during the whole life-cycle, they do include exposure during the most sensitive life-stages, and have shown to predict accurately maximum acceptable toxicant concentration (MATC) values (comprised in the range defined by the NOEC and LOEC) in fish early life-stage tests.

Emission mapping from different industries can be helpful and technology is instrumental in detecting possible new pollutants. However, during their pass through wastewater treatment plants, and before reaching the final receiving waters, pollutants can suffer different physico-chemical processes such as biodegradation or biotransformation which could vary estimated final concentrations. It may be more time sparing and effective to measure final concentrations at discharge points and in final receiving environments. It is in this context that advanced technology is necessary for detection of possible new contaminants. In any case, knowledge of real, local contaminant concentrations would make environmental management independent from industry information, which, in combination with the knowledge of $LC_{50}$ values of the most sensitive components of the concrete environment, would be sufficient to manage the most important contaminants.

The objective of this work is to analyse the toxicity of different LAS homologues and commercial LAS on larvae of the marine fish *Sparus aurata* and to examine the environmental implications of these pollutants in coastal marine ecosystems affected by wastewater. The results corresponding to the effects of LAS exposure on seabream eggs have been published recently in Hampel and Blasco[12] and are cited in this context in order to compare differences in sensitivity to surfactant exposure at different development stages. Early life-stage toxicity tests may be useful for the evaluation of the impact of pollutants in coastal marine ecosystems.

## EXPERIMENTAL METHODS

The assays were carried out following the procedures proposed by the OECD[13] and the EPA[14].

Fertilised seabream eggs were maintained under controlled laboratory conditions until hatching and 25 neonate larvae, not older than 24 h, were transferred into 1 l glass recipients containing LAS (homologues or commercial LAS) dissolved in pure filtered sea water (0.45 μm) at concentrations between 0.01 and 10.0 mg $l^{-1}$, and exposed during 72 h in a thermostatic chamber at constant temperature (22 ± 1ºC) and photoperiod 12 h light/12 h darkness. Soft aeration was guaranteed during the whole assay duration. LAS concentrations in the reference water were not detectable with the employed techniques (<1 μg $l^{-1}$). Exposition to the different concentrations was realised in triplicate and three control assays were performed simultaneously with pure filtered seawater. Nominal concentrations were assumed to be constant as the exposure solution was renewed completely each 24 h, which is lower than the half-life for LAS under aerobic conditions in sea water[6].

Dead and living larvae were counted and reported after every 24 h and dead organisms were removed from the recipients. The employed mortality criterion was the complete loss of heartbeat, which is generally combined with the loss of flotability and the appearance of a white coloration. $LC_{50}$ values as well as standard deviation were calculated and compared with $LC_{50}$ (24 h) values from previous hatching experiments with eggs of the same species[15]. Dividing these obtained values with average representative homologue concentrations[16], potentially hazardous homologues can be detected by extrapolating the ratio $LC_{50}$ (24 h)/environmental concentration with different security factors proposed by TEGEWA, EU, and ECETOC[17]. If this ratio is higher than the factor proposed, present homologue concentration is not considered as hazardous for the existing population. Additionally, differences in sensitivity between both development stages, eggs, and larvae were detected by calculation of the ratio $LC_{50}$ (eggs)/$LC_{50}$ (larvae).

All statistical analysis were performed using the program INSTAT (GraphPad Software). Analysis of variance (ANOVA) was performed in order to determine statistical differences between means. When occurring significant differences between mean values, the Tukey test ($p <$ 0. 05) was used to determine significant differences between means.

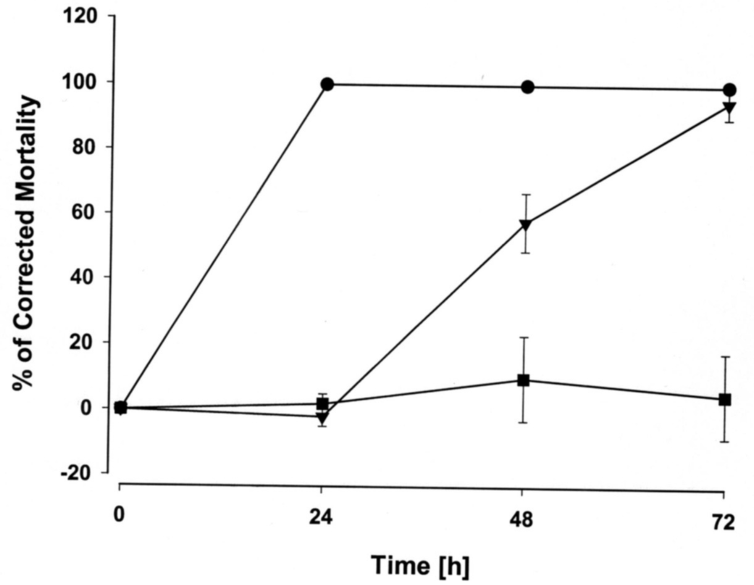

**FIGURE 1.** Percentages of corrected mortality at three different exposure concentrations for LAS homologue $C_{14}$ vs. exposure time. (● = 0.2 mg $l^{-1}$; ▲ = 0.1 mg $l^{-1}$; ■ = 0.07 mg $l^{-1}$).

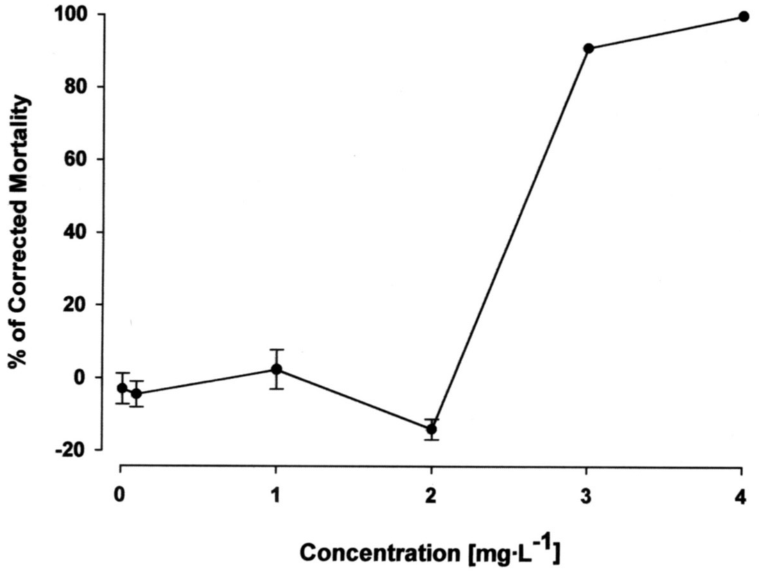

**FIGURE 2.** Percentages of corrected mortality of the seabream, *Sparus aurata,* vs. exposure concentration for LAS homologue $C_{11}$ after 24 h of exposure.

## RESULTS AND DISCUSSION

Mortality of exposed larvae at relevant LAS concentrations depends on both surfactant concentration and exposure time. Fig. 1 shows the percentages of accumulated mortality vs. time for the homologue LAS $C_{14}$ at three different concentrations as an example for larvae mortality as a consequence of LAS exposure. Fig. 2 shows the accumulated mortality percentages for the

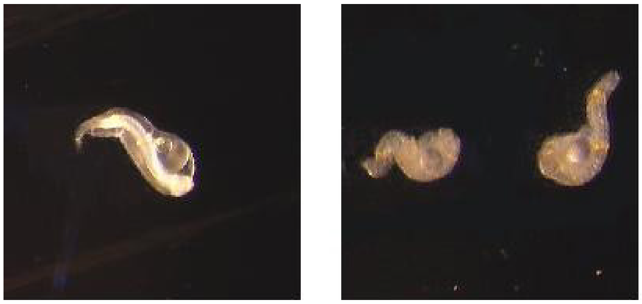

**FIGURE 3.** Seabream larvae exposed to lethal LAS concentrations. (a) Larva exposed to 0.25 mg $l^{-1}$ LAS $C_{14}$; (b) larva exposed to to 0.50 mg $l^{-1}$ LAS $C_{14}$.

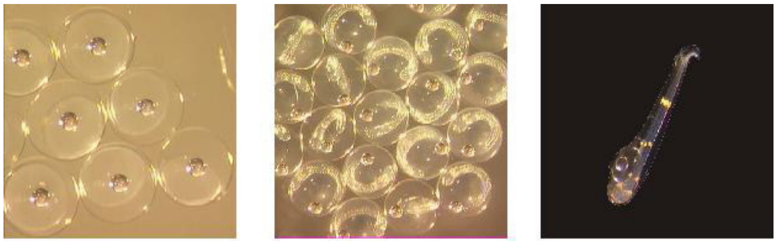

**FIGURE 4.** Control eggs before (a) and after (b) gastrulation, as well as control larva (c) of the seabream, *Sparus aurata*.

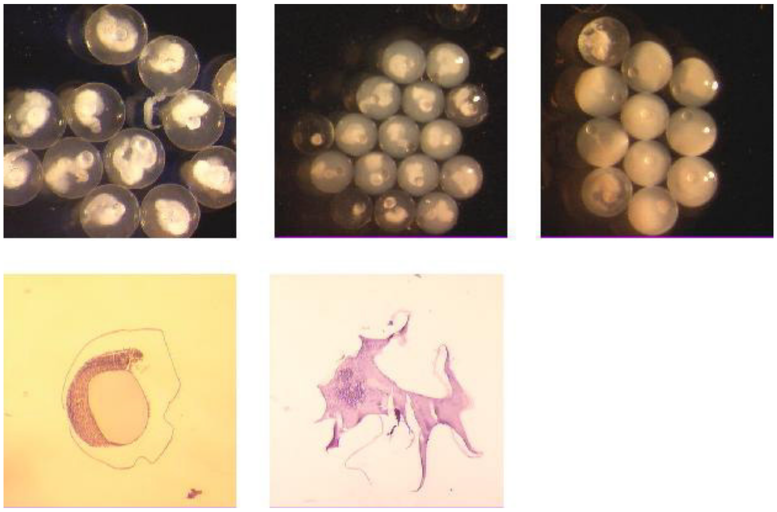

**FIGURE 5.** Seabream eggs after exposure to increasing LAS concentrations. (a,b,c) Eggs after exposure to 0.25, 0.50, and 5.00 mg $l^{-1}$ LAS $C_{13}$, respectively. (d,e) Histopathological view of seabream eggs after exposure to 5.00 and 10.0 mg $l^{-1}$ LAS $C_{10}$, respectively.

homologue LAS $C_{11}$ after 24 h with the chosen increasing surfactant concentrations. At lethal concentrations, dead larvae with white coloration can be observed at the bottom of the vessel. This white coloration increases in intensity the higher are the lethal surfactant concentrations (Fig. 3a,b), while the larvae and eggs from the control assays are normally transparent and flote inactively in the water column (Fig. 4a-c). In hatching experiments with eggs from the same species realised previously[14], dead eggs also showed increasing white coloration at increasing lethal concentrations (Fig. 5a-e).

**TABLE 1**
**$LC_{50}$ values obtained in Larvae Exposure Assays with Different LAS Homologues after 24, 48, and 72 h of Exposure**

| LAS Homologue | $LC_{50}$ (24h) | $LC_{50}$ (48h) | $LC_{50}$ (72h) |
|---|---|---|---|
| LAS $C_{10}$ | - | 3.7 ± 0.2 | 2.6 ± 0.3 |
| LAS $C_{11}$ | 3.0 ± 0.3 | 2.9 ± 3.0 | 2.0 ± 1.2 |
| LAS $C_{12}$ | 0.2 ± 0.1 | 0.2 ± 0.0 | 0.1 ± 0.1 |
| LAS $C_{13}$ | 0.1 ± 0.0 | < 0.1 | < 0.1 |
| LAS $C_{14}$ | 0.2 ± 0.0 | 0.2 ± 0.0 | 0.1 ± 0.0 |
| Commercial LAS | 0.7 ± 0.1 | 0.5 ± 0.0 | 0.5 ± 0.0 |

**TABLE 2**
**Homologue Composition of the Employed Commercial LAS Mixture**

| Homologue distribution | Percentage (%) |
|---|---|
| < Phenyl $C_{10}$ | 1.1 |
| Phenyl $C_{10}$ | 10.9 |
| Phenyl $C_{11}$ | 35.3 |
| Phenyl $C_{12}$ | 30.4 |
| Phenyl $C_{13}$ | 21.2 |
| Phenyl $C_{14}$ | 1.1 |
| 2 Phenyl Alkane | 15.5 |

Source: PETRESA

$LC_{50}$ values after 24, 48, and 72 h were calculated (Table 1) from the obtained mortality data. The tendency, observed previously by other authors[18,19,20,21], that the toxicity of LAS homologues increases with increasing alkyl chain length which is reflected in lower $LC_{50}$ values was confirmed. For example, the $LC_{50}$ (24 h) values for each LAS homologue and commercial LAS, with an average alkyl chain length of 11.6 carbon atoms (Table 2), range between concentrations of 0.1 and 3.0 mg $l^{-1}$ corresponding the higher limit to the shorter (LAS $C_{10}$) and the lower to the longer homologues (LAS $C_{14}$). The longer the alkyl chain of the homologues, the more lipophilic is their behaviour, which facilitates the interaction with biological tissues[9]. In both cases — eggs and larvae (Table 3), the $LC_{50}$ values decrease exponentially when increasing the number of carbon atoms in the alkyl chain, resulting in an important decrease of the $LC_{50}$ value between the homologues $C_{11}$ and $C_{12}$. This fact implies an important increase of the toxicity exerted on the exposed organisms of homologues with 12 or more carbon atoms in their

**TABLE 3**
**$LC_{50}$ Values and Standard Deviation after 24 h of Exposure to Different LAS Homologues and Commercial LAS for Seabream Eggs and Larvae, As Well As Ratio between $LC_{50}$ (Eggs) and $LC_{50}$ (Larvae)**

| Compound | $LC_{50}$ (24h) [$mg{\cdot}L^{-1}$] ± SE Eggs | $LC_{50}$ (24h) [$mg{\cdot}L^{-1}$] ± SE Larvae | $LC_{50}$(eggs)/$LC_{50}$(larvae) |
|---|---|---|---|
| LAS $C_{10}$ | 4.4 ± 1.2 | - | - |
| LAS $C_{11}$ | 7.7 ± 0.8 | 3.0 ± 0.3 | 2.6 |
| LAS $C_{12}$ | 0.6 ± 0.6 | 0.2 ± 0.1 | 3.0 |
| LAS $C_{13}$ | 0.1 ± 0.0 | 0.1 ± 0.0 | 1.0 |
| LAS $C_{14}$ | 0.2 ± 0.0 | 0.2 ± 0.0 | 1.0 |
| Comercial LAS | 0.5 ± 9.5 | 0.7 ± 0.1 | 0.7 |

alkyl chain. Commercially employed LAS is a mixture of LAS homologues with alkyl chain length between 10 and 14 carbon atoms at different proportions and has an average alkyl chain length of 11.6 carbon atoms. Its $LC_{50}$ value of 0.7 mg $l^{-1}$ is more similar to that obtained for LAS $C_{12}$ than for LAS $C_{11}$. In any case, toxicity seems to increase exponentially (exponential decrease of $LC_{50}$ values) when adding a carbon atom to the alkyl chain.

The $LC_{50}$ (24 h) values from the larvae exposure experiments were compared with the results obtained in previous hatching experiments with eggs of the same species, *Sparus aurata*[12] by calculating the ratio between both (Table 3), making possible a comparison of the sensitivities at both development stages, egg and larva. The ratio obtained for each homologue was plotted vs. number of carbon atoms in the alkyl chain (Fig. 3). Using this ratio between $LC_{50}$ (24) values from hatching experiments with eggs and the realised exposure experiments with larvae, it can be observed that larvae are up to 3.0 times more sensitive to LAS exposure than eggs (Table 3) during the first 24 h of exposure. This difference in sensitivity decreases when increasing the number of carbon atoms in the alkyl chain of the homologues, which is reflected in the progressive decrease of this ratio. Nevertheless, the ratio obtained for commercial LAS of 0.7 indicates that in this case, eggs were more sensitive to LAS exposure than larvae and does not show a behaviour similar to LAS $C_{12}$ as would be expected for an average chain length of 11.6 carbon atoms.

The main reason for the higher sensitivity of larvae to pollutant exposure is the fact that the transport rate of xenobiotics across the chorion is lower compared to the rate of transport across the gill epithelium of the larvae. The final penetration of the pollutants into the embryo tissues is probably lower than in larvae for two reasons: eggs have a smaller surface area exposed to the environment than larvae, and eggs do not actively circulate fluid through or near the chorion while larvae circulate blood through the gills[22]. The decrease of the ratio between $LC_{50}$ (24) values of eggs and larvae means that the sensitivity of the exposed development stages to LAS exposure is more similar for the longer homologues than for the shorter which implies that longer homologues interact more easily with the embryo and, hence they have more facility to penetrate the chorion. When increasing the number of carbon atoms in the alkyl chain, the hydrophobic character of the homologue also increases. It is possible that this fact facilitates the penetration of the surfactant into the egg.

Considering the ratio between $LC_{50}$ value and the concentration of the corresponding homologue in an environment which could be considered as representative for untreated

**TABLE 4**
**Environmental Concentrations of the Different LAS homologues in the Sancti Petri Channel (San Fernando, Spain), $LC_{50}$ (24h) Values for Eggs and Larvae, As Well As the Ratio between Environmental Concentration and Obtained $LC_{50}$ (24 h) Values**

| LAS Homologue | Env. Concentration [$\mu gL^{-1}$] | $LC_{50}$ (eggs) [$\mu gL^{-1}$] | $LC_{50}$ (larvae) [$\mu gL^{-1}$] | Ratio $LC_{50}$/Env. Concentration (eggs) | Ratio $LC_{50}$/Env. Concentration (larvae) |
|---|---|---|---|---|---|
| $C_{10}$ | 2 | 4400 | - | 2200 | - |
| $C_{11}$ | 8 | 7700 | 3000 | 1026 | 375 |
| $C_{12}$ | 5 | 600 | 200 | 134 | 40 |
| $C_{13}$ | 1 | 100 | 100 | 100 | 100 |
| $C_{14}$ | - | 200 | 200 | - | - |

wastewater discharge of a population of about 100 000 inhabitants, the safety criteria proposed by the mentioned organisms are not always fulfilled in the case of the longer homologues. LAS $C_{12}$ and $C_{13}$ homologue concentrations are considered as harmful by the EU and ECETOC for eggs and larvae. For the larvae, the ratio between environmental concentration and $LC_{50}$ for LAS $C_{12}$ is 40, close to the limit to be considered safe even for the criterion proposed by TEGEWA (Table 4).

## CONCLUSIONS

In the case of the marine fish, *Sparus aurata*, the larvae development stage has shown to be more sensitive towards surfactant exposure than the egg. Nevertheless, this difference in sensitivity depends on the hydrophobicity of the different homologues. In the case of the longer homologues, eggs and larvae present similar responses, which is related to the increasing hydrophoic character of these compounds. For this reason, the recommended development stage for toxicity testing is the larvae, due to its mayor sensitivity towards surfactant exposure, as the survival of every development step of an organism is necessary in order to guarantee the integrity and therefore the persistance of the ecosystem.

Even if early life-stage toxicity tests provide useful information about lethal environmental concentrations in form of $LC_{50}$ values, histopathological research is restricted as internal malformations may manifest after advanced development which could have consequences in combination with environmental stress or provoke a less resistant second generation. Nevertheless, employment of cytohistochemical techniques such as evaluation of activity of antioxidant enzymes, peroxisomal proliferation, and lipid peroxidation can provide important additional information about the effects of a surfactant containing environment.

## ACKNOWLEDGEMENTS

This work has been supported by the Environmental and Climate Program of the European Commission PRISTINE (Contract No: ENV4-CT97-494) in the framework of the Wastewater Cluster. The paper was presented at the CSIC/ESF workshop Analysis, Toxicity and Biodegradation of Organic Pollutants in Groundwater from Contaminated Land, Landfills and

Sediments, Barcelona, Spain, 8–10 November 2001. We wish to thank the company PETRESA for the LAS supply and financial support. M. Hampel was supported by a fellowship of the CSIC.
